# Indomethacin induced gene regulation in the rat hippocampus

**DOI:** 10.1186/s13041-015-0150-x

**Published:** 2015-10-06

**Authors:** Monica Sathyanesan, Matthew J. Girgenti, Jennifer Warner-Schmidt, Samuel S. Newton

**Affiliations:** Division of Basic Biomedical Sciences, Sanford School of Medicine, University of South Dakota, Vermillion, SD 57069 USA; Department of Psychiatry, Yale University School of Medicine, New Haven, CT 06519 USA; Laboratory of Molecular and Cellular Neuroscience, Rockefeller University, New York, NY 10065 USA

**Keywords:** NSAID, Hippocampus, Cyclooxygenase inhibitor, Gene expression, Cerebrovascular

## Abstract

**Background:**

Non-steroidal anti-inflammatory drugs such as indomethacin are widely used to treat inflammatory diseases and manage pain, fever and inflammation in several conditions, including neuropsychiatric disorders. Although they predominantly function by inhibiting cyclooxygenase (COX) activity, important COX-independent actions also occur. These actions could be responsible for the adverse side effects associated with chronic and/or high dose usage of this popular drug class.

**Results:**

We examined gene regulation in the hippocampus after peripheral administration of indomethacin by employing a microarray approach. Secondary confirmation and the brain expression pattern of regulated genes was examined by *in situ* hybridization and immunohistochemistry. Transglutaminase 2, serum glucocorticoid inducible kinase, Inhibitor of NF-kappa B and vascular endothelial growth factor were among genes that were prominently upregulated, while G-protein coupled receptor 56 and neuropeptide Y were among genes that were downregulated by indomethacin. Co-localization studies using blood vessel markers revealed that transglutaminase 2 was induced specifically in brain vasculature.

**Conclusions:**

The data demonstrate that COX-inhibitors can differentially regulate gene transcription in multiple, functionally distinctly cell types in the brain. The results provide additional insight into the molecular actions of COX-inhibitors and indicate that their effects on vasculature could influence cerebral blood flow mechanisms.

## Introduction

Pharmacological inhibitors of cyclooxygenases (COX), non-steroidal anti-inflammatory drugs (NSAIDs), have been widely used for several decades as anti-pyretics, anti-inflammatory agents and analgesics. They function by inhibiting the COX enzymes, COX-1 and/or COX-2 which are responsible for the production of prostaglandins and mediating inflammation. As inflammation has been implicated in the etiology of several neurodegenerative illnesses it supports the testing of NSAIDs for neuroprotective and therapeutic effects [1, 2]. However, the results of large scale clinical testing in Alzheimer’s disease patients have indicated that contrary to the expectations NSAIDs had an adverse rather than a beneficial effect [3]. Furthermore, clinical studies have shown that NSAID use is associated with a higher risk for stroke [4]. Large scale studies of NSAID use by healthy individuals have uncovered significantly elevated incidence of cardiovascular events and stroke [5].

Prostaglandin synthesis is known to drive the expression of growth factors such as basic fibroblast growth factor (bFGF) and vascular endothelial growth factor (VEGF), molecules that play key roles in angiogenesis [6]. NSAIDs are therefore prescribed in the treatment of cancer [7] for their anti-angiogenic and anti-proliferative effects with the intention of suppressing tumors by depriving them of vascular support and inhibiting cell proliferation [8]. Despite the wide therapeutic applications of NSAIDs, the risk of cardiovascular and cerebrovascular effects has led to elevated caution in their use [9]. Employing transgenic receptor knockout mice, the notably higher cardiovascular risk associated with highly selective COX-2 inhibitors was attributed to their specific inhibition of prostacyclin, a vasodilator, but allowing COX-1 mediated thromboxane A2, a vasoconstrictor, to produce unopposed prothrombotic effects [10]. However, detailed meta-analysis of randomized human trial data found the incidence of cardiovascular events in the use of COX-2 specific inhibitors to be similar to that of conventional NSAIDs [11]. It is also not understood how peripherally administered NSAIDs influence the risk of vascular complications in the brain.

To obtain insight on the molecular actions of a non-selective NSAID on the brain we tested indomethacin, which inhibits both COX-1 and COX-2. We examined gene expression changes in the hippocampus using a focused microarray after peripheral administration in rats. Gene expression changes were independently validated by quantitative PCR, *in situ* hybridization and immunohistochemical analysis. Indomethacin induced regulation of trophic factor levels were measured in the plasma and brain.

## Results

### Indomethacin-induced gene regulation

**Fig. 1:**
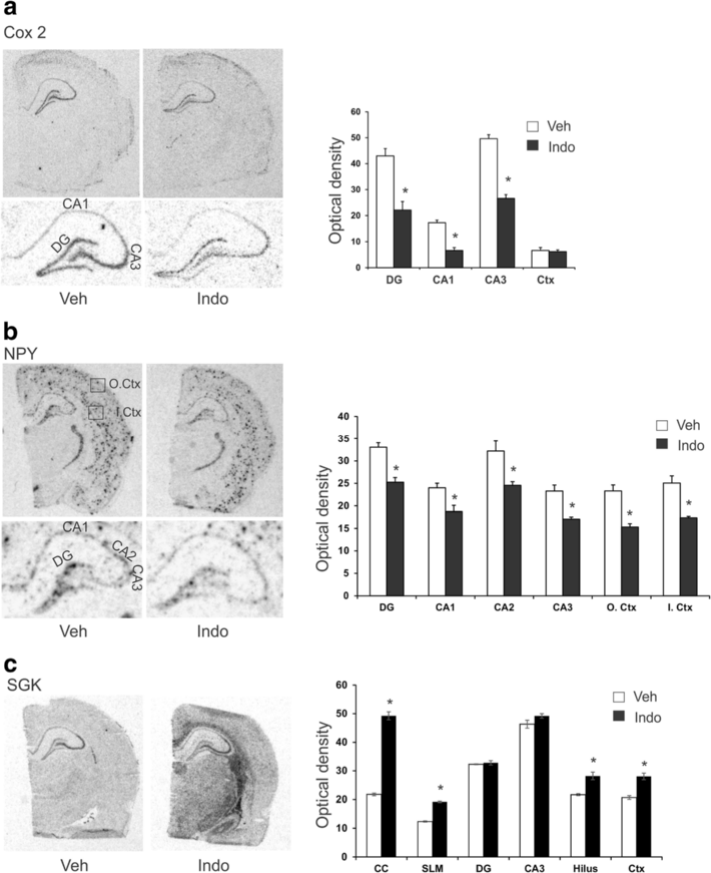
*In situ* hybridization analysis of indomethacin-induced gene regulation. Representative photomicrographs of hippocampal sections hybridized with radiolabeled riboprobes. Quantified expression of the indicated layers is shown by bar graphs on the right. Results are expressed as optical density values relative to vehicle treatment and are the mean ± SEM of four separate animals, each analyzed in duplicate brain sections. **a** Downregulation of Cox-2 is prominently seen in the hippocampus but not the cortex. Hippocampal cell layers are identified in the lower panel, showing reduction in the dentate gyrus (DG), CA1 and CA3 regions. **b** NPY downregulation is evident in all the brain regions examined, hippocampus and cortex. Expression in the inner and outer cortical layers were quantitate using boxes. **c** Marked SGK upregulation is seen in the white matter corpus callosum (CC) and lower levels of induction in the *stratum lacunosum moleculare* (SLM), hilus and cortex

**Fig. 2:**
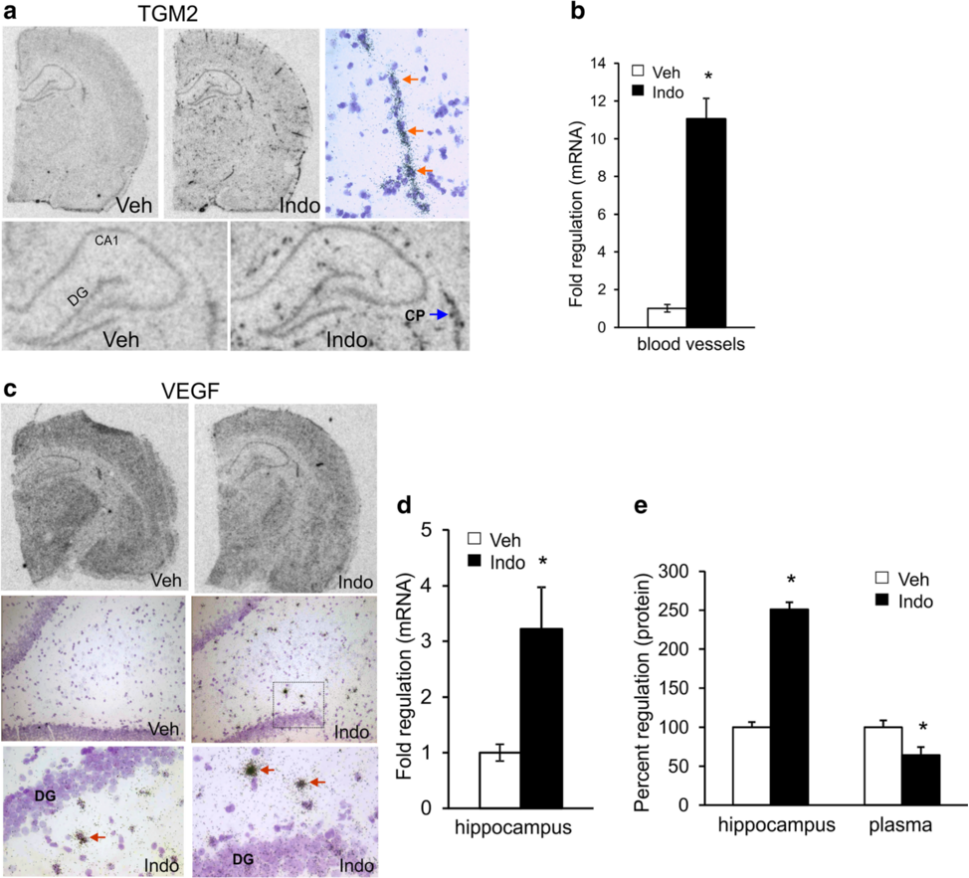
Regulation of TGM2 and VEGF. **a** *In situ* hybridization (*left* and *middle* panel) and emulsion autoradiography (*right* panel) photomicrographs of TGM2 induction are shown in hippocampal section. The hippocampus is shown at higher magnification in the *lower* panels. Striking induction in TGM2 is seen in multiple brain regions, including the choroid plexus (CP) after indomethacin administration. Emulsion autoradiographs are representative images from indomethacin treated brains. The vascular expression pattern is indicated by the colored arrows. **b** TGM2 induction in isolated cortical blood vessels was measured by QPCR. *Bar graphs* represent the mean from 3 animals, *error bars* are SEM (*p* < 0.05). **c** VEGF expression in vehicle and indomethacin treated animals is shown in the *top* panel. Cresyl violet stained emulsion autoradiographs of vehicle and indomethacin treated animals is shown in the *middle* panel. The boxed region in the indomethacin treated section is shown at two levels of magnification in the *bottom* panel. **d** VEGF induction in the hippocampus was examined by QPCR. Bar graphs represent the mean from 3 animals, error bars are SEM (*p* < 0.05). **e** ELISA analysis of VEGF protein levels in the hippocampus and plasma. VEGF in indomethacin treated animals are shown as a percentage of vehicle VEGF levels. *Bar graphs* represent the mean from 4 animals, *error bars* are SEM (*p* < 0.05)

Employing a focused microarray that included 3000 genes representing trophic factor signaling and transcription factor genes we examined the gene profile of a non-selective Cox inhibitor, indomethacin, in the adult rat brain. Peripheral administration was sufficient to alter gene regulation in the hippocampus. Indomethacin increased the expression of 14 genes and decreased the expression of 6 genes (Table 1). Six genes were robustly induced, exhibiting an upregulation higher than 2-fold. *In situ* hybridization analysis showed that gene expression changes were not limited to the hippocampus as several genes, NPY, SGK, TGM2 and VEGF were also regulated in the cortex (Figs. 1, 2). Cox-2 mRNA was downregulated specifically in the dentate gyrus, CA1 and CA3 layers and did not change in the cortex (Fig. 1a). NPY, which exhibited a punctate expression pattern, decreased in all the regions of the hippocampus and also the inner and outer cortical layers (Fig. 1b). Serum glucocorticoid-inducible kinase (SGK) was strikingly elevated in white matter regions and only mildly elevated in the cortex. Increase in hippocampal SGK was limited to the *stratum lacunosum moleculare* (SLM) and hilus (Fig. 1c).

**Table 1:** List of indomethacin regulated genes in the rat hippocampus is shown with corresponding GenBank accession number, fold regulation and *P*-value

Gene name	Accession number	Fold regulation	P-value
UPREGULATED GENES			
Metallothionein 2	NM 001137564.1	3.12	0.001
Serum glucocorticoid inducible kinase	NM 019232.3	3.07	0.017
Transglutaminase 2	NM 019386.2	2.79	9.05E-07
NF-kappa-B inhibitor alpha	NM 001105720.2	2.77	0.003
S100A9-S100 calcium binding protein A9	NM 053587.1	2.76	0.002
SIN3A-SIN3 transcription regulator A	NM 001108761.1	2.74	7.53E-06
Y box binding protein 3	NM 031979.3	1.98	2.68E-06
VEGF	NM 031836.3	1.92	0.025
Numbl	NM 001033888.1	1.85	0.016
Prefoldin 4	XM 003749636.2	1.84	3.93E-04
FGF2	NM 019305.2	1.72	0.006
MKP1-MAP kinase phosphatase 1	AF357203.1	1.63	0.004
Endothelial Nitric oxide synthase 3	CAB 77547.1	1.61	0.002
Hes 1-hes family bHLH transcription factor 1	NM 024360.3	1.59	0.001
DOWNREGULATED GENES			
Zbtb20-zinc finger and BTB domain 20	NM 001105880.1	0.37	0.011
G-protein coupled receptor 56	NM 152242.2	0.55	0.001
Milk fat globule EGF-8	NM 012811.3	0.59	0.005
FGF9	D14839.1	0.62	0.033
Squalene synthase	M95591.1	0.64	0.005
Neuropeptide Y	NM 012614.2	0.67	0.01

Data are from *N* = 4 and *P* < 0.05

Transglutaminase 2 (TGM2) was expressed at low levels in the brain of vehicle treated rats (Fig. 2a). Although TGM2 was strongly induced by indomethacin the somewhat punctate and “hot spot” expression pattern did not appear restricted to the hippocampus. Cortical induction suggested high levels of expression in blood vessels (Fig. 2a). Emulsion autoradiography provided additional support to vascular expression of TGM2 as emulsion grain density revealed a vascular pattern. Furthermore, quantitative PCR analysis of isolated cortical vessels showed a 10-fold higher expression in indomethacin treated rats compared to controls (Fig. 2b).

**Fig. 3:**
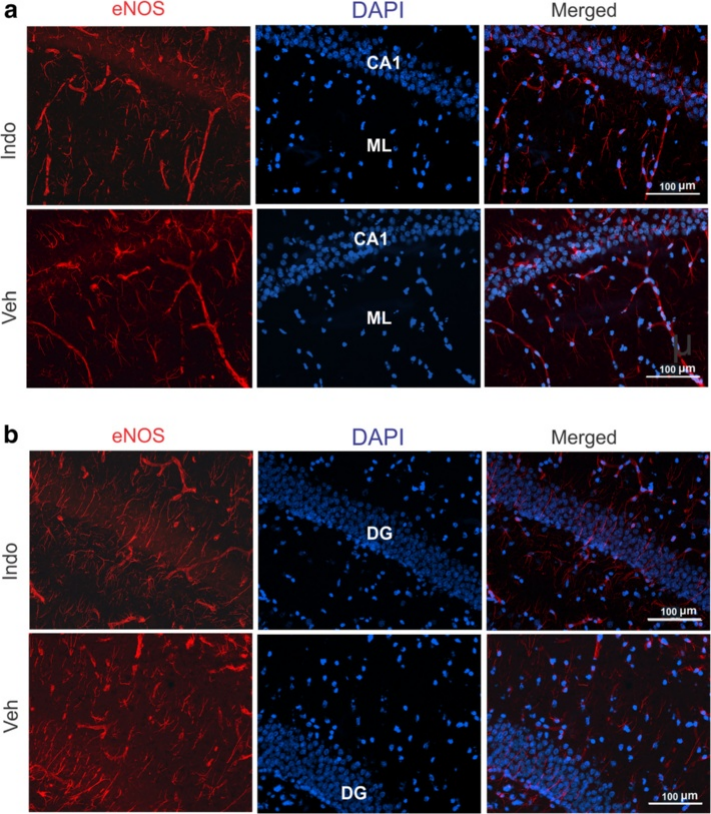
Immunohistochemical analysis of eNOS expression in the brain. **a** eNOS expression in the hippocampal CA1 and molecular layer (ML) is shown after indomethacin and vehicle administration. **b** eNOS expression in the dentate gyrus cell layer is shown after indomethacin and vehicle administration

*In situ* hybridization analysis of vascular endothelial growth factor (VEGF) did not reveal any clearly discernible elevation in mRNA (Fig. 2c). However, quantitative PCR demonstrated a 3-fold upregulation of VEGF (Fig. 2d), which was higher than the 1.9 fold regulation indicated by the array experiments (Table 1). We therefore performed emulsion autoradiography to obtain cell-level resolution of VEGF expression. As shown in the two bottom panel images of Fig. 2c (lower magnification at left and higher magnification on the right) VEGF was highly induced in particular cells in the hilus. Cells in the dentate gyrus did not exhibit this high level of VEGF expression. ELISA Immunoassay analysis of VEGF protein reflected the mRNA regulation observed in the hippocampus (Fig. 2e). It is interesting to note that in contrast to VEGF elevation in the hippocampus, indomethacin decreased VEGF in the plasma (Fig. 2e), albeit at lower levels. Array analysis indicated a modest upregulation in eNOS mRNA. eNOS expression was therefore examined using immunohistochemistry (Fig. 3). Expression appeared to be in the vasculature and astrocytic processes, but eNOS protein did not show appreciable indomethacin-induced upregulation that was observed at the mRNA level.

### TGM2 expression and cellular colocalization

**Fig. 4:**
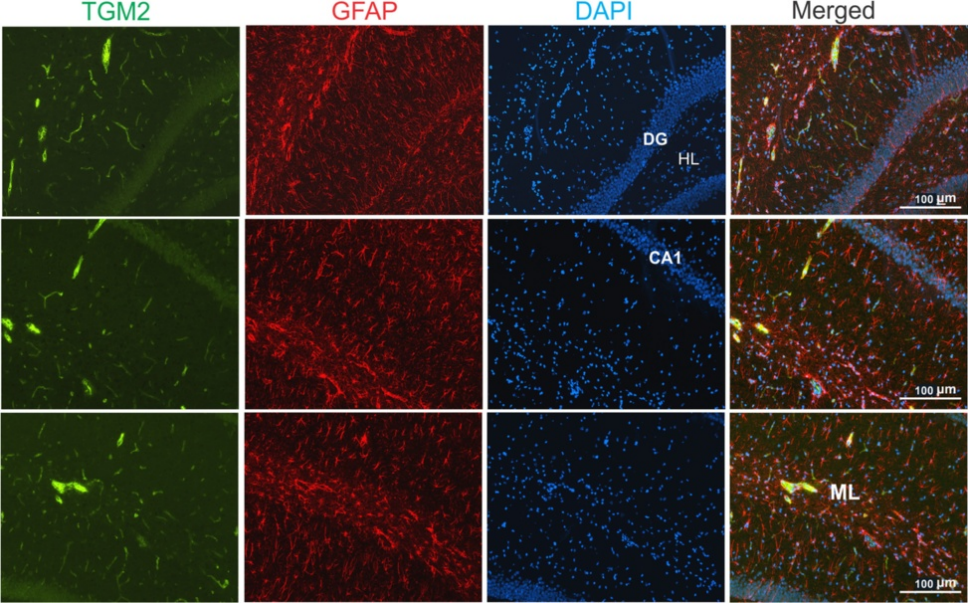
Astrocytic localization of indomethacin-induced TGM2. Colocalization analysis of GFAP and TGM2 in the hippocampal cell layers is shown. Merged images combining GFAP, TGM2 and nuclear DAPI staining is shown in the *right-most* panel. HL- hilus, ML- molecular layer, DG – dentate gyrus

**Fig. 5:**
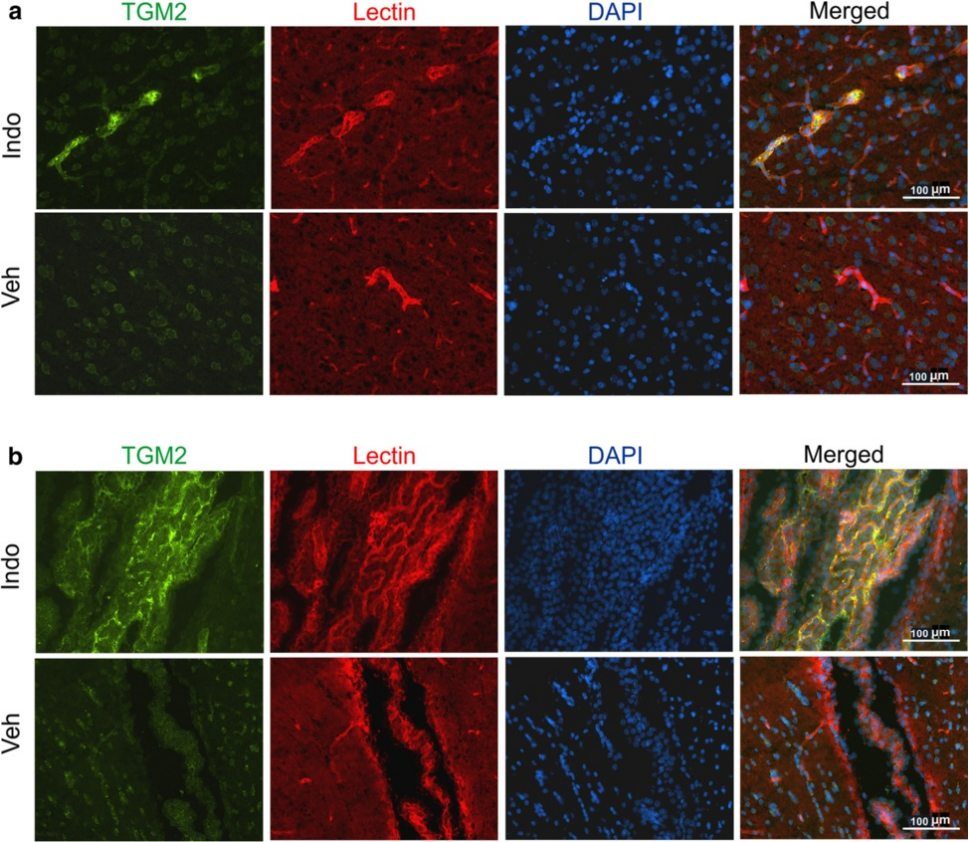
Immunohistochemical analysis of TGM2 induction in the brain. **a** TGM2 expression in the discrete cortical blood vessels is shown. Fluorescent-tagged tomato lectin was used a blood vessel marker and DAPI was used to label nuclei. Indomethacin treated sections are in the *top row* and vehicle treated in the *bottom row*. Merged images of all 3 channels are in the *right-most* panel. **b** Strong TGM2 expression is shown in the lateral choroid plexus. Indomethacin treated sections are in the *top row* and vehicle treated in the *bottom row*. Merged images of all 3 channels are in the *right-most* panel

**Fig. 6:**
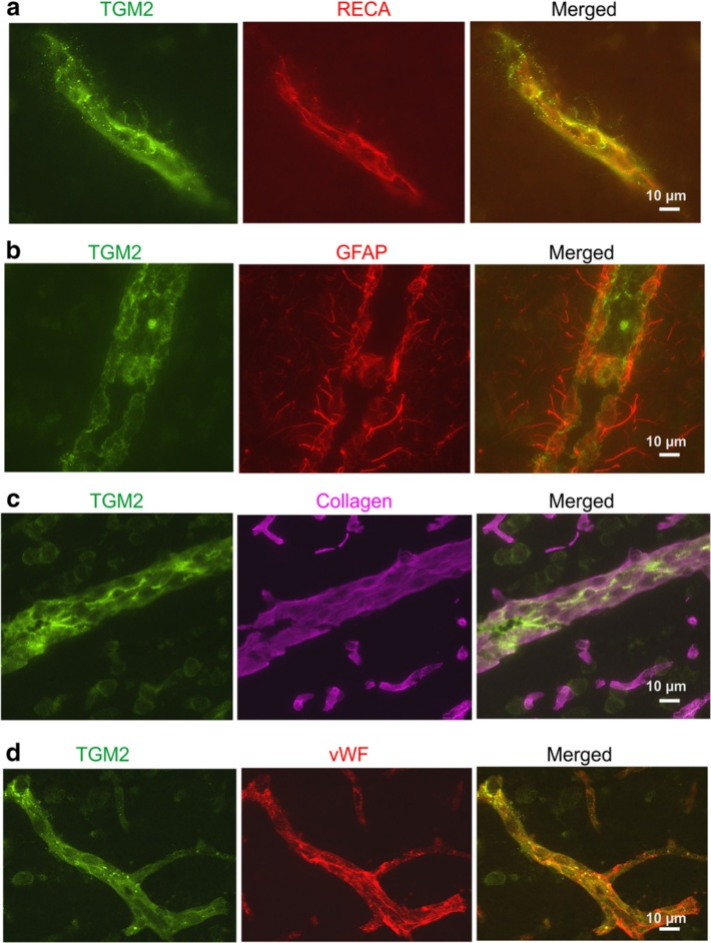
Co-localization analysis of indomethacin induced TGM2 expression in cortex blood vessels. **a** TGM2 expression in endothelial cells is shown using rat endothelial cell antigen (RECA) as an endothelial marker. **b** Using GFAP as an astrocytic marker shows that TGM2 is not expressed in GFAP-positive astrocytes. **c** TGM2 does not co-localize with Collagen IV which was used as basement membrane marker. **d** TGM2 expression in endothelial cells is shown using Von willebrand factor (vWF) as an additional endothelial marker

Astrocytic expression of TGM2 protein was examined in the hippocampus using glial fibrillary acidic protein (GFAP) as the marker (Fig. 4). Although TGM2 was expressed close to astrocytic processes, no colocalization was observed, suggesting that astrocytes are not a site of TGM2 production. Immunohistochemical analysis showed that TGM2 was expressed at low levels in dentate gyrus neurons (Fig. 4) and that the indomethacin-induced upregulation was mainly in the vasculature (Fig. 5a). Blood vessels were identified by staining with fluorescently labeled tomato lectin. TGM2 expression in blood vessels was not uniform and exhibited punctate regions of high expression. We also noted that the highest levels of TGM2 expression and indomethacin-induced elevation was in larger vessels (>10 μm in diameter). Neuronal TGM2 expression was comparable in both indomethacin and control groups. Strong induction of TGM2 occurred in the choroid plexus and appeared specific to CP blood vessels and not the epithelial cells (Fig. 5b). To obtain further insight into the vascular expression of TGM2 we performed immunohistochemical analysis using additional cell type markers (Fig. 6). Rat endothelial cell antigen (RECA), a widely used marker of endothelial cells, revealed substantial colocalization with TGM2 (Fig. 6a). However, grains representing high levels of TGM2 appeared to be extracellular. We tested the role of the basement membrane by utilizing collagen IV as a specific marker. Although collagen IV yielded a clear vascular pattern, its expression did not colocalize with TGM2 (Fig. 6c). Von willebrand factor staining colocalized with TGM2 and provided evidence that endothelial cells are the likely source of indomethacin-induced vascular upregulation (Fig. 6d).

### Cox2 specific inhibitor induces vascular TGM2

**Fig. 7:**
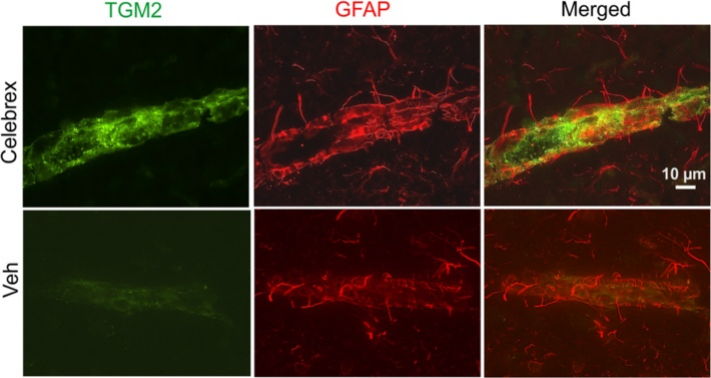
Celebrex-induced TGM2 expression in cortical blood vessels is shown. Low levels are detectable in vehicle treated animals. Expression does not co-localize with GFAP-positive astrocytes and appear to be in endothelial cells. Merged images combining TGM2 and GFAP channels are in the *right* panel

As indomethacin is a nonselective inhibitor of cyclooxygenase we investigated a selective COX-2 inhibitor, celecoxib, for its ability to regulate TGM2 expression. Celecoxib strongly induced TGM2 in cerebral blood vessels (Fig. 7). The expression pattern of Celecoxib-induced TGM2 was similar to indomethacin, larger cortical vessels. Double immunohistochemical analysis with GFAP showed that, like indomethacin-induced TGM2, celecoxib-induced TGM2 was also not expressed in GFAP-positive astrocytes. Overall, the level of TGM2 induction appeared to be higher with celecoxib administration in comparison to the indomethacin induced elevation.

## Discussion

We conducted a gene expression analysis in rat brain tissue after peripheral NSAID administration. Although our focus was the hippocampus, subsequent secondary validation was performed using *in situ* hybridization and immunohistochemical analyses, yielding regional and cellular insight.

NSAIDs are considered safe medications and therefore highly used worldwide to treat a variety of pain and inflammation conditions. They are also used long term at high doses as antitumor agents [12]. As several NSAIDs are available over-the-counter, the potential for chronic usage or in large doses is particularly high. While the predominant mechanism attributed to their actions is the inhibition of prostaglandin synthesis, NSAIDs have also been shown to produce a spectrum of useful effects that are cyclooxygenase and prostaglandin-independent [13]. For example, the ability to inhibit angiogenesis has attractive clinical applications in cancer treatment [14], but require doses that are several fold higher than those used to inhibit prostaglandins [13]. These antitumorigenic and antiangiogenic doses have been demonstrated to directly alter gene expression in different cell types [15, 16], suggesting that NSAIDs possess multiple modes of action.

Our brain gene expression study revealed several target genes that have not been previously associated with NSAID treatment. While NSAID induced metallothionein gene regulation has been previously reported in the liver, our array experiments show that it produces a similar increase in the hippocampus. Metallothionein induction in the brain has been implicated in neuronal repair mechanisms and occurs as a protective response to injury and inflammation [17]. They are secreted by reactive astrocytes and modulate intracellular actions via the neuronal megalin receptor. Elevating brain MT levels via NSAID administration can protect against neuroinflammation and also elevate trophic factors such as erythropoietin which has wide spectrum neuroprotective effects. Serum and glucocorticoid-inducible kinase (SGK) is induced in the brain primarily by glucocorticoids [18], but also by several different stimuli and stressors, including fear conditioning, enriched environment, plus maze exposure, cell volume and drugs of abuse [19]. However, our *in situ* hybridization analysis shows that the highest levels of regulation are in white matter regions. This could be in response to indomethacin-induced reduction in blood flow. SGK is induced in the hippocampus and corpus callosum after transient global ischemia [20] and serves to protect against apoptosis by activating PI3K/Akt signaling. The high sensitivity of white matter to ischemia is well known [21] and the accompanying cell volume changes could be the likely trigger for SGK upregulation as an alteration in cell volume is a strong stimulus for its induction [19].

The topic of indomethacin-induced ischemia has been controversial as human imaging studies have demonstrated the known vasoconstrictor effects of indomethacin [22] but no ischemic regions were observed [23]. Studies in pigs [24] and baboons [25] have shown marked decreases in cerebral blood flow, lowering it by 30 % in these species. The possibility of microvascular ischemia has not been sufficiently explored and could be challenging for live imaging analysis as it would require capillary level resolution. The robust vascular induction of transglutaminase 2 (TGM2) is perhaps the most interesting outcome of this study. TGM2 is a multifunctional, scaffolding, calcium-dependent enzyme that is expressed at low levels in the brain but is strongly induced after stroke injury [26, 27]. TGM2 overexpression increases cell death while inhibitors and gene deletion produce beneficial effects [27, 28]. However, it appears that cellular phenotype is a critical factor as TGM2 deletion in neurons renders them vulnerable to ischemic stress while ablation in astrocytes is protective [28]. Our co-localization experiments indicate that TGM2 induction occurs in endothelial cells and not in neurons or astrocytes.

Secreted protein TGM2 can act on cells in the vicinity to elicit diverse functions. It is sharply elevated by vasoconstriction and plays a central role in the inward remodeling of small arteries following a reduction in blood flow [29]. Blood vessels are held in the constricted state by a specific TGM2 activity that cross-links several extracellular matrix proteins such as collagen, fibronectin and vitronectin, resulting in lasting vascular entrenchment and reduced diameter [30, 31]. The array data indicated a modest induction in eNOS. This is intriguing as eNOS is known to inhibit the crosslinking activity of TGM2 via nitric oxide mediated *S*-nitrosylation [32]. eNOS could also produce vasodilation to counter some of the vasoconstrictive actions of TGM2. However, we were unable to detect eNOS induction at the protein level. It is likely that the mild induction of eNOS mRNA does not result in detectable levels of eNOS protein elevation. The upregulation of TGM2 in the choroid plexus (CP) could influence CSF secretion. Blood flow in the CP is 3 times faster than other brain regions [33] in order to facilitate its secretory functions. The CP plays important roles in brain homeostasis and function [34, 35]. If TGM2 constricts CP capillaries and reduces blood flow it is likely to negatively impact CSF production and overall CP function.

Indomethacin is clinically effective in decreasing elevated intracranial pressure caused by head injury, and is mediated by decreasing cerebral blood flow [36]. The molecular mechanism involved in this action is not currently understood. Our data indicate that TGM2 could be involved in the vascular effects of indomethacin. As both celecoxib and indomethacin similarly induced TGM2 it is likely that it is a consequence of their ability to modulate gene transcription rather than COX inhibition. Alternately, it could be related to COX-2 inhibition as both indomethacin and celecoxib suppress COX-2. It is interesting to note the downregulation of GPR56, an atypical G-protein coupled receptor that binds TGM2 and antagonizes its activity [37]. In the brain, GPR56 is abundantly expressed in the endfeet of radial glia and regulates basement membrane integrity [38].

The differential effects on VEGF levels in plasma versus the hippocampus is intriguing. The lowering of plasma VEGF was expected due to the known anti-angiogenic activity of NSAIDs, but the brain upregulation appears paradoxical. A previous indomethacin study reported a hypoxia-induced elevation of VEGF and FGF2 in the renal cortex [39]. The increase in hippocampal VEGF could be due to localized hypoxia that is detected by particular astrocytic cells that respond by upregulating VEGF mRNA [40]. NF-kappa B (NF-κB) is an inducible transcription factor that serves as a key mediator of inflammation by regulating the expression of several downstream pro-inflammatory genes. NSAIDs, particularly salicylates such as Aspirin, are known to inhibit NF-κB and do so by suppressing the degradation of its inhibitor, IκBα, via inactivation of the kinase IKK [41]. The induction of IκBα gene expression that we observed appears to be an additional transcriptional mechanism for inhibiting NF-κB.

The downregulation of Neuropeptide Y (NPY) in the cortex and hippocampus by indomethacin could have negative behavioral consequences under stressful conditions as the NPY system plays an important role in countering the adverse emotional and behavioral effects of stress [42–44]. NPY is induced in the hippocampus by antidepressant treatment [45, 46] and independently produces antidepressant [47] and anxiolytic [48] effects. Although adverse behavioral consequences associated with NSAID use are rare, indomethacin and selective COX-2 inhibitors have been reported to exacerbate symptoms in patients suffering from psychiatric illnesses [49]. This gene expression study sheds light on the potential CNS effects of NSAIDs and also provides an explanation for some of their observed vascular actions. As NSAIDs constitute 5–10 % of all drug prescriptions and are frequently used chronically, future studies should focus on lower dose but longer treatment regimens and also seek to clarify the cyclooxygenase-dependent and independent effects.

## Conclusions

In summary, we conclude that COX inhibitors elicit gene expression changes in the brain and produce differential cell-type specific effects by acting on neuronal and vascular cells. The elevation in vascular TGM2 could potentially impact cerebrovascular tone and blood flow. Our results provide new insight into the molecular actions of COX inhibitors and their ability to influence gene transcription in the hippocampus.

## Methods

### Animals and treatment

Male SD rats (weighing 250–275 g) were pair-housed and maintained in standard conditions with a 12-h light/dark cycle and *ad libitum* access to food and water. All animal use procedures were in strict accordance with the National Institutes of Health *Guide for the Care and Use of Laboratory Animals* and were approved by the Animal Care and Use Committee. Indomethacin, Celecoxib (Cayman Chemical Company) or vehicle (10 % DMSO in phosphate buffered saline) was injected (10 mg/kg, i.p.) once daily for three days. Animals were sacrificed 7 h after their last injection. The hippocampus was dissected, rapidly frozen on dry ice and kept at −80 °C until used. Trunk blood was collected into vacutainer tubes containing sodium heparin (BD Biosciences). Blood was centrifuged at 1000 × G for 15 min at 4 °C. Plasma aliquots were stored at −80 °C prior to use.

### Protein detection

Hippocampus and plasma levels of VEGF (R&D Systems) protein were assessed by ELISA kit according to manufacturer’s instructions. Whole hippocampus was homogenized in 0.5 ml buffer (137 mM NaCl, 20 mM Tris–HCl (pH 8.0), 1 % NP40, 10 % glycerol) and 50 μl of homogenate was used for each assay. Samples were run in duplicate. Total protein concentration was determined by BCA assay according to manufacturer’s instructions (Pierce Biotechnology, Inc., Rockford, IL).

### Microarray analysis of gene expression

Array analysis was performed using a focused microarray platform [50]. The hippocampus was manually dissected and quickly frozen on dry ice. RNA was isolated using a non-phenolic procedure (RNAqueous kit, Ambion). RNA yields were quantitated by Nanodrop spectrophotometer (Thermo Scientific) and quality was verified by gel electrophoresis or real time PCR. 3ug of hippocampal RNA was reverse transcribed into cDNA using oligo dT primers and indirectly labeled using fluorescent dendrimers (Genisphere, Hatfield, PA). A two-step hybridization and labeling protocol was used where the chip was hybridized to cDNA overnight, washed and post-stained with fluorescent dendrimers. Slides were scanned using a GenePix scanner (Molecular Devices, Sunnyvale, CA). Image analysis was performed using GenePix Pro software. Resulting files were imported into GeneSpring (Agilent Technologies, Santa Clara, CA) for additional visualization and data mining. A gene was considered as being expressed if its signal intensity was a minimum of twice the background in 3 of the 4 replicates. Per-chip normalization was performed by dividing the expressed genes by the median of two housekeeping control genes that were not regulated, b-tubulin and cyclophilin. Gene regulation was determined by taking the log ratio of the median experimental channel signal to the median control channel signal. Upregulated genes were defined as having an average expression ration of >1.3, and the downregulated genes were defined as having an average expression ratio of <0.7. These values were determined by performing homotypic hybridizations where the same sample was hybridized in both channels (cy3 and cy5).

### Statistical analysis

Analysis of microarray data was performed by an unpaired *t* test using the cross-gene pooled error method in Genespring software. Relative gene expression using quantitative PCR was calculated using the ΔΔCt method and analyzed using the *t* test in GraphPad software. *In situ* hybridization images were quantified using NIH Image software, and statistical analysis (*t*-test) was performed using Microsoft Excel Analysis tool Pak.

### *In situ* hybridization and emulsion autoradiography

ISH was performed using radiolabeled riboprobes according to conventional protocols with minor modifications [51]. Riboprobes were generated by PCR amplification using gene-specific primers, Cox2 - CGCTGCTGCCGGACACCTTC, CCACCAGCAGGGCGGGATAC; VEGF – TGCCCACGTCGGAGAGCAAC, CTCAAGCTGCCTCGCCTTGC. The reverse primer included a T7 template sequence (TAATACGACTCACTATAGGGAGA) on the 5′end. Whole rat brain cDNA was used as the template for PCR, which was performed in a real-time PCR instrument using the Quantitect Sybr Green PCR kit (Qiagen). PCR product was purified by ethanol precipitation and resuspended in TE buffer. One microgram of the 300–400 bp PCR product was used to produce radiolabeled riboprobe using a T7-based in vitro transcription kit (Megashortscript; Ambion, Austin, TX, USA). All riboprobes were verified by sequencing of the PCR product.

### Real-time PCR analysis

Gene specific primers for TGM2, VEGF, FGF2, NFKBIA, SGK, S100A9 were designed using “Primer 3” web-based software. Amplification parameters were 15 min at 95 °C (to activate hotstart Taq polymerase) and then 94 °C 2 s, 60 °C 30 s and 72 °C 30 s. PCR quantitation was performed by converting differences in Ct (cycle threshold) values to fold regulation after normalizing to the unregulated housekeeping gene, cyclophilin. Blood vessels were manually isolated from the outer surface of the brain and placed in eppendorf tubes that were then frozen on dry ice. Blood vessel isolation was performed with care to minimize any inclusion of surrounding neural tissue. RNA from blood vessel and hippocampus tissue were used to make cDNA for comparison of transcript levels of TGM2 in both tissue types.

### Immunohistochemistry

Immunohistochemical studies were performed using cryocut coronal sections (16 μm). Sections were fixed in Histochoice fixative (Sigma) or cold 4 % formalin for 10 min followed by a 5 min wash in cold 1x PBS. All incubations were performed on the slide by encircling sections with an ImmEdge pen (Vector Laboratories, Burlingame, CA, USA), then adding antibody solution directly to the sections. Sections were blocked with 5.0 % BSA (w/v) in PBS (Molecular biology grade lyophilized bovine serum albumin, Sigma) at 4 °C for 30 min. Sections were incubated with different primary antibody combinations in antibody solution (2.5 % BSA in PBS) at 4 °C overnight (TGM2, 1:150, Cell Signaling (Beverly, MA, USA); Lectin-Dylight 594 (1:500, Vector Laboratories, Burlingame, CA, USA); RECA (1:50, Serotec (Oxford, UK); GFAP (1:1000), Collagen IV (1:100, Abcam, Cambridge, MA, USA), eNOS (1:300, Abcam). Following primary antibody incubation, slides were washed in 1xPBS three times for 5 min each at room temperature. Slides were then incubated in appropriate fluorescent secondary antibody (1:500, Alexa-488 and 594, Life Technologies) in 2.5 % BSA in PBS for 2 h at room temperature. The slides were then rinsed in 1x PBS three times for five minutes each and coverslipped using VectaMount (Vector Labs). Sections were viewed and images were captured using a Nikon Eclipse Ni microscope equipped with DS-Qi1 monochrome, cooled digital camera and NIS-AR 4.20 Elements imaging software. Indomethacin, and vehicle treated sections were captured using identical exposure settings.
